# An Array Magnetic Coupling Piezoelectric and Electromagnetic Energy Harvester for Rotary Excitation

**DOI:** 10.3390/mi14081527

**Published:** 2023-07-29

**Authors:** Qiuxuan Chen, Chong Li, Mingming Lv

**Affiliations:** School of Mechanical Engineering, Jiangsu University of Science and Technology, Zhenjiang 212100, China

**Keywords:** energy harvester, piezoelectric macro fiber composite (MFC), magnetic coupling, output power

## Abstract

The energy of rotating machinery exists widely in the environment. It is of great significance to collect and utilize the energy of rotating machinery for sustainable development. In this paper, a novel piezoelectric and electromagnetic energy harvester, which is capable of generating electrical energy under rotary excitation, is proposed based on array magnetic coupling. The working principle of this kind of energy harvester is analyzed. And the energy output modeling of the harvester is developed and output results are simulated. Based on the experimental test platform built in the laboratory, the output characteristics of the piezoelectric and electromagnetic energy harvester are tested. Results show that the maximum output power of the proposed energy harvester reaches 182 mW when the excitation speed is 120 rpm. Furthermore, both the piezoelectric module and the electromagnetic module can reach the maximum output power at the excitation speed of 120 rpm.

## 1. Introduction

With the rapid development of society, its demand for energy has greatly increased [1,2]. In the face of the dual challenges of global environmental degradation and the energy crisis, countries around the world have introduced low-carbon policies and actively explored green and clean energy [3,4,5]. As a result, various types of energy-harvesting devices have been developed for generating electric power from renewable energies like solar, wind, ocean wave, and so on [6,7,8].

In industrial production and life, rotational mechanical energy of is abundant in the environment, and it can be easily converted into electric energy with the help of the corresponding external excitation conversion equipment. A substantial amount of research has been conducted in order to find the best energy-harvesting mechanisms, such as electromagnetic, electrostatic, piezoelectric, and triboelectric [9,10,11,12].

Among the energy-harvesting mechanisms mentioned above, electromagnetic, piezoelectric, and triboelectric are the main research objects in the current research. Although current researchers have conducted numerous studies on the materials, structures, and applications of energy harvesters, the most advanced single-mechanism energy harvesters still feature the problem of limited power. To overcome the limited output power generated by an energy harvester, researchers have tried mixing two or more energy conversion mechanisms. Commonly used hybrid energy harvesting methods include electromagnetic and triboelectric [13,14,15], piezoelectric and triboelectric [16,17,18], piezoelectric and electromagnetic [19,20,21]. However, in the case of triboelectric, due to the need for perfect contact and separation between the two friction layers to enhance power output, energy-harvesting devices using triboelectric components have a larger displacement compared to other hybrid-mechanism types of energy-harvesting devices, which limits the energy-harvesting device to operating at low frequencies. There is no frequency or acceleration limitation for energy acquisition from piezoelectric and electromagnetic mixtures. It is easy to produce high power output at low acceleration and medium and high frequency without excessive use of mechanical parts. In addition, piezoelectric and electromagnetic materials have the advantages of high power density, high conversion efficiency, and strong adaptability. Therefore, compared with the mixing of several other mechanisms, the piezoelectric–electromagnetic hybrid form is more suitable in practice.

A lot of research has been conducted on piezoelectric and electromagnetic energy harvesters and many results have been achieved. Han et al. [22] designed a 2 × 2 array piezoelectric–electromagnetic hybrid energy harvester. The harvester included four piezoelectric–electromagnetic hybrid modules, each of which consisted of a piezoelectric sheet, a permanent magnet, and a wound coil. Jiang et al. [23] proposed a novel low-frequency hybrid piezoelectric and electromagnetic broadband harvester. The design, analysis, and experiment of the designed energy harvester were carried out. The results show the maximum power of the harvester is 6.28 mW. To realize the harvesting of energy generated by human movement, a piezoelectric–electromagnetic composite was designed for harvesting the energy generated by low-frequency human motion by Zhang et al. [24]. The maximum output voltage of the harvester is 25.6 V at the step frequency of 140 steps/min. Moreover, to enlarge the frequency bandwidth and obtain a larger energy output, a piezoelectric–electromagnetic energy harvester was designed by Shan et al. [25]. This proposed harvester consists of a primary piezoelectric energy harvesting device, in which a suspension electromagnetic component was added. The results show that the experimental results are consistent with the theoretical analysis and finite element simulation results. Furthermore, a vibration energy harvester that couples piezoelectric effect and electromagnetic induction was proposed based on polyvinylidene fluoride [26]. The harvester realizes the expected functions of inputting alternating current and outputting direct current. Through experimental tests, the open circuit output voltage and output power are up to 6.55 V and 2.21 mW. In the field of energy harvesting, many new materials have been developed for energy harvesting. Yan et al. [27] studied the porous ferroelectric materials used for energy technologies. Through the introduction of porosity into dense ferroelectric materials, the performance of piezoelectric and pyroelectric has been improved. Deng et al. [28] introduced and analyzed the piezoelectric nano-generators for personalized healthcare. As a typical smart material, a 3–3 piezoelectric composite has a wide application in the fields of ultrasonic transducer, underwater acoustic detection, and so on. Li et al. [29] investigated a water-based PZT slurry of 52.0 vol% solid content with high storage, loss modulus, and shear thinning characteristics. The results demonstrated that the 3–3 piezoelectric composite has great potential for tuning and optimizing the piezoelectric properties of piezocomposites. To achieve wearable physiological monitoring, Su et al. [30] developed a muscle-fiber-inspired, nonwoven piezoelectric textile with tunable mechanical properties. The piezoelectric textile demonstrates multiple potential applications, including pulse wave measurement, human motion monitoring, and active voice recognition.

For the piezoelectric–electromagnetic hybrid energy harvester under rotary excitation, numerous achievements have been made. Using force amplification mechanisms, Zhao et al. [31] developed a novel water-proof hybrid wind energy harvester. The key components of the energy harvester can be packaged easily, owing to the non-contact magnetic coupling mechanism. Hence, the proposed energy harvester can work in a harsh environment. Additionally, the harvester can collect saturates at 3157.7 mu W with a wind speed of 7.0 m/s under rainfall. To collect the energy from falling water, He et al. [32] proposed a hybrid energy harvester that combined piezoelectric and electromagnetic mechanisms. Two piezoelectric energy harvesters and two electromagnetic energy harvesters work together to improve the output characteristic. The research results showed that the maximum output power of the proposed harvester is 22.78 mW. Wang et al. [33] designed a hybrid piezoelectric–electromagnetic energy harvester driven by contactless rotary magnetic plucking. The effects of the driving force parameters, load resistance, and electromechanical coupling strengths on the maximal displacements and velocities, average power inputs and outputs, energy efficiencies of the system are investigated. The hybrid energy harvester can achieve higher power outputs than the single piezoelectric and the single electromagnetic energy harvester.

Although significant progress has been made, it still remains a challenge to gather the electrical energy under the rotary excitation. In this paper, a novel piezoelectric–electromagnetic hybrid energy harvester, which is capable of generating electrical energy under the rotary excitation, is developed based on array magnetic coupling. The proposed energy harvester combines piezoelectric power generation technology and electromagnetic power generation technology. The energy harvester has the advantages of large output energy of the electromagnetic power generation, a simple structure, and no electromagnetic interference of the piezoelectric power generation. Because of the array arrangement of piezoelectric plates, magnets, and coils in the structure, the designed energy harvester can achieve a high output power. Compared with other energy harvesters, the hybrid energy harvester proposed in this paper realizes stable output of higher power, and the maximum output power reached 182 mW. The output power is much higher than that of the same type of energy harvester.

The working principle of the proposed energy harvester is analyzed. The energy output modeling of the harvester is established, and the output voltage and power of the harvester is simulated. Additionally, the performance of the prepared hybrid harvester is evaluated via a home-made experimental platform.

## 2. Design

**Figure 1:**
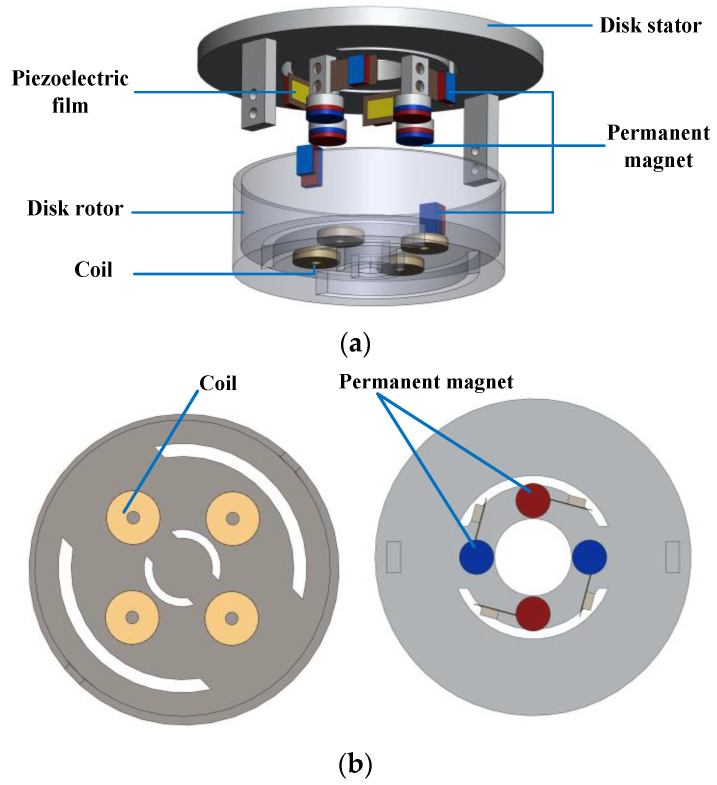
Structure of the piezoelectric–electromagnetic hybrid energy harvester. (**a**) 3D exploded drawing; (**b**) Partial structure drawing.

Figure 1 shows the structure of the proposed hybrid energy harvester, which is mainly composed of a piezoelectric power generation module and an electromagnetic cooperation module.

The piezoelectric power generation module is mainly composed of piezoelectric macro fiber composite (MFC) films, a disk stator, and permanent magnets. The piezoelectric MFC material has the advantages of good flexibility, large deformation, and long service life. When the energy harvester is working, the piezoelectric cantilever beam will produce large deformation, so the MFC material is selected in this paper to achieve piezoelectric power generation. The specific characteristics and parameters of MFC piezoelectric films are listed in Table 1. The piezoelectric MFC films are attached to the cantilever beams, the fixed ends of which are connected to a static disk stator. Since there is sufficient distance between two adjacent cantilever beams, there is no magnetic interaction between the permanent magnets at the free end of the cantilever beams. 

**Figure 2:**
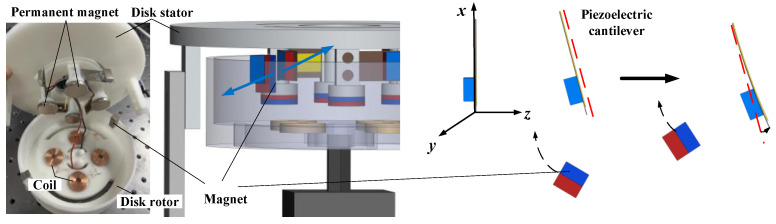
Principle of piezoelectric cantilever beam deformation under magnetic force.

The working principle of the disk rotor driving the cantilever beam deformation, as shown in Figure 2, the permanent magnet is embedded in the disk rotor, and the magnet at its free end is excited when it is near the cantilever beam. The direction of piezoelectric cantilever beam compression is the direction of *z* axis. The horizontal direction along the plane where the piezoelectric plate located is the *x* axis direction, and the *y* axis direction is the vertical downward direction of the plane where the piezoelectric plate located. Due to the repulsion of the same polarity, the cantilever beam deflects to the right, and the piezoelectric film produces bending deformation and generates electric energy. When the permanent magnet moves away from the cantilever, the cantilever returns to its original state and continues to vibrate. In addition, the deformation of the cantilever beam depends on the electromagnetic force, and the piezoelectric MFC film deformation is converted into electrical energy while keeping the electromagnetic force in a reasonable range to maintain the normal continuous rotation of the rotor. To this end, the magnetic force provided by the permanent magnet cannot be too small to ensure the vibration amplitude of the cantilever beam, and the magnetic force cannot be too large to prevent the repulsion force from being too large to complete the periodic rotation.

The parameters of the electromagnetic power module are shown in Table 2. The electromagnetic cooperative module is composed of coils, permanent magnets, a disk rotor, and a conductive slip ring, which converts kinetic energy of rotor into electric energy via electromagnetic induction. To generate electricity through electromagnetic effect, four coils are embedded in the bottom of the disk stator, the same numbers of permanent magnets are installed in the stator part opposite the coil, the adjacent polarity is alternating. The coil is wound with copper wire, and the magnet material is an NdFeb high-strength magnet. All coils are connected in a series to obtain relatively large internal resistance. When the disk rotor rotates, the permanent magnet placed on the rotor begins to rotate, the piezoelectric module begins to work, the coil placed on the rotor also begins to rotate, and the electromagnetic cooperation module begins to work under the action of the magnet installed in the stator and the adjacent polarity alternating. As an important part of the piezoelectric power generation module, the conductive slip ring is connected to the coil placed on the disk rotor to solve the problem of the coil winding in the process of following the rotor in the 360° periodic rotation, which affects the system performance and energy measurement, so as to ensure the stable and accurate transmission of current and signal.

## 3. Energy Output Modeling and Simulation Analysis

The piezoelectric module generates voltage through the deformation of the piezoelectric MFC films. When the deformation of the piezoelectric MFC film is *δ_p_*, the output current of the load for the piezoelectric generator is

(1) $$i_{p}\left(t\right)=\frac{\partial Q\left(t\right)}{\partial t}=d_{31}ES_{p}\frac{\partial \delta_{p}\left(t\right)}{l_{p}\partial t}$$

where *d*_31_ is piezoelectric strain constant, *E* is Young’s modulus of MFC film, *S_p_* is the surface area of the piezoelectric material, *l_p_* is the length of piezoelectric MFC film.

The output power of the piezoelectric module can be written as

(2) $$P\left(t\right)=\frac{Q^{2}\left(t\right)}{2C}=d_{31}^{2}E^{2}S_{p}^{2}\frac{\delta_{p}^{2}\left(t\right)}{2Cl_{p}^{2}}$$

where *C* is the capacitance of piezoelectric MFC.

The rotation between the coil and the permanent magnet generates an induced current, the permanent magnet on the stator is stationary, and the coil rotates with the rotor. In a closed circuit, both current and electromotive force are present. The electromotive force produced in electromagnetic phenomena can be expressed as:

(3) $$\left\{\begin{array}{l} E=N\frac{d\phi }{dt}\\ \phi =BS\cos\varpi_{1}+\varpi_{2}t\\ \theta =\theta_{0}+\varpi_{1}+\varpi_{2}t \end{array}\right.$$

where *N* is the number of turns, ϕ is the rate of flux change in a closed circuit; *B* represents the magnetic field strength and *S* represents the actual area of the coil; *θ*_0_ and *θ* are the initial phase angle and rotation angle of the magnet; *ω*_1_ and *ω*_2_, respectively represent the angular velocity of the S-type rotor.

The voltage generated by the rotating magnetic field can be written as

(4) $$U=E=(R_{coil}+R_{load})i_{coil}$$

where *R_coil_* and *R_load_* are the internal resistance of the coil and the load resistance, *i_coil_* is the coil current.

The power output of a load resistor can be expressed as

(5) $$P_{load}={\left(\frac{U}{R_{coil}+R_{load}}\right)}^{2}R_{load}={\left(12N\varpi \frac{d\phi }{(R_{coil}+R_{load})d\theta }\right)}^{2}R_{load}$$

In the case of *R_coil_* being equal to *R_load_*, the maximum output power of the electromagnetic module is easily obtained as

(6) $$P_{\max}=\frac{12}{R_{load}}{\left(\frac{N\varpi d\phi }{d\theta }\right)}^{2}$$

**Figure 3:**
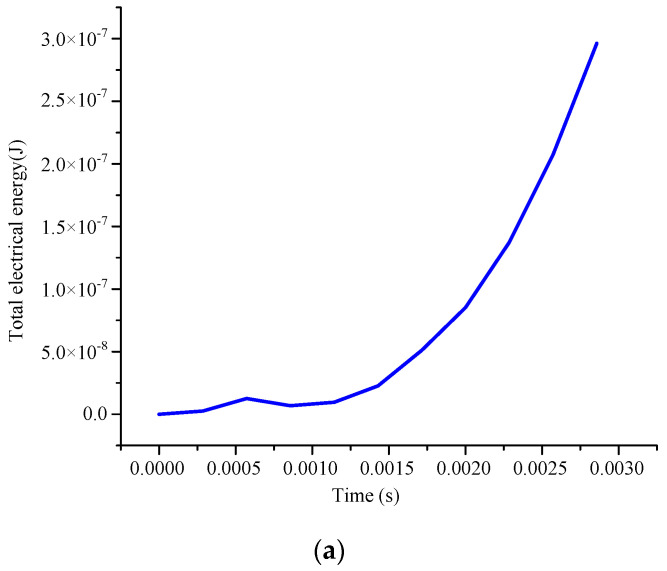

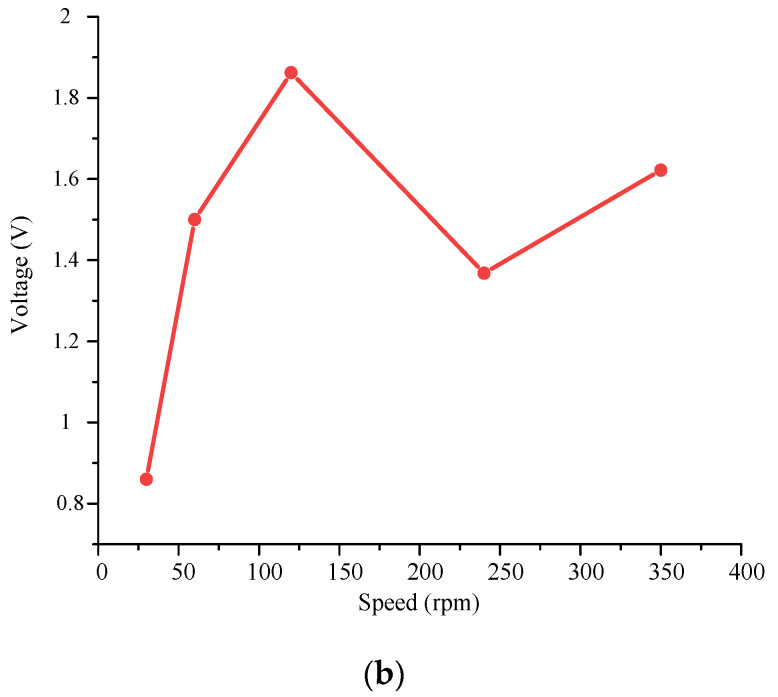
Simulation result of the output voltage of the piezoelectric MFC film. (**a**) Total electrical energy changes with time; (**b**) voltage changes with exciting rotary speed.

To verify the design scheme of the energy harvester in this paper, COMSOL 6.1 software is used to simulate the energy harvester. Figure 3 shows the simulation result of the output voltage of the piezoelectric MFC film. Figure 3a presents the total electrical energy changes with time in a cycle. In one cycle, the magnet will gradually move closer to the piezoelectric MFC film and then gradually move away. In this process, the electromagnetic force received by the piezoelectric cantilever will gradually reach its peak from a small value. In this paper, the change in electric energy generated by piezoelectric plates is simulated at 30 rpm. By applying the rotating electromagnetic force approximated as a sine wave to the piezoelectric MFC film for simulation, the total electric energy generated by the piezoelectric effect can be gradually increased within a certain time range. From Figure 3b, it can be seen that when the driving speed is less than 120 rpm, the output voltage increases with the increase of the speed. When the driving speed is greater than 120 rpm, the output voltage fluctuates and shows a downward trend. Meanwhile, the output voltage of the piezoelectric module reaches the maximum value of 1.87 V when the excitation speed is 120 rpm.

**Figure 4:**
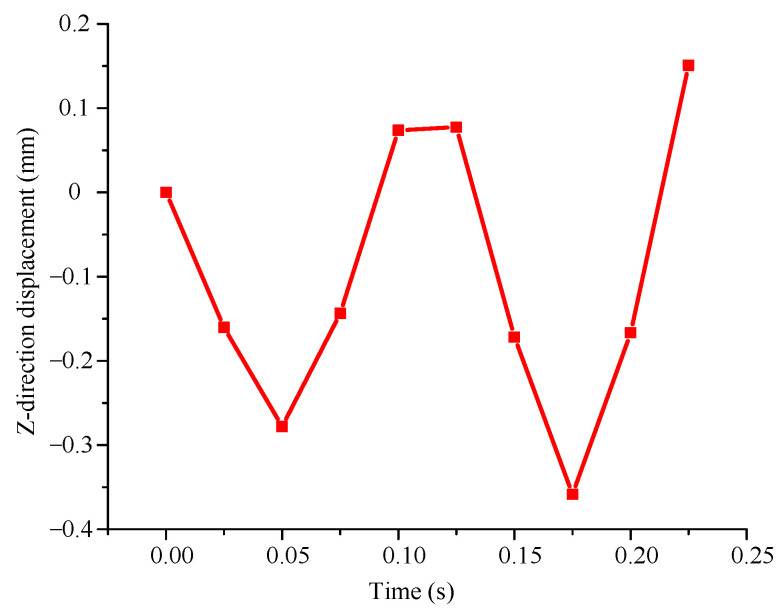
Displacement of the piezoelectric MFC film changes in the *z* direction.

To analyze the displacement change of piezoelectric cantilever beam under the action of magnetic force, the output displacement of piezoelectric cantilever beam is simulated. Figure 4 shows the displacement of the piezoelectric MFC film changes in the *z* direction. The results show that the piezoelectric MFC film vibrates periodically along the *z* axis, which is consistent with the original design.

**Figure 5:**
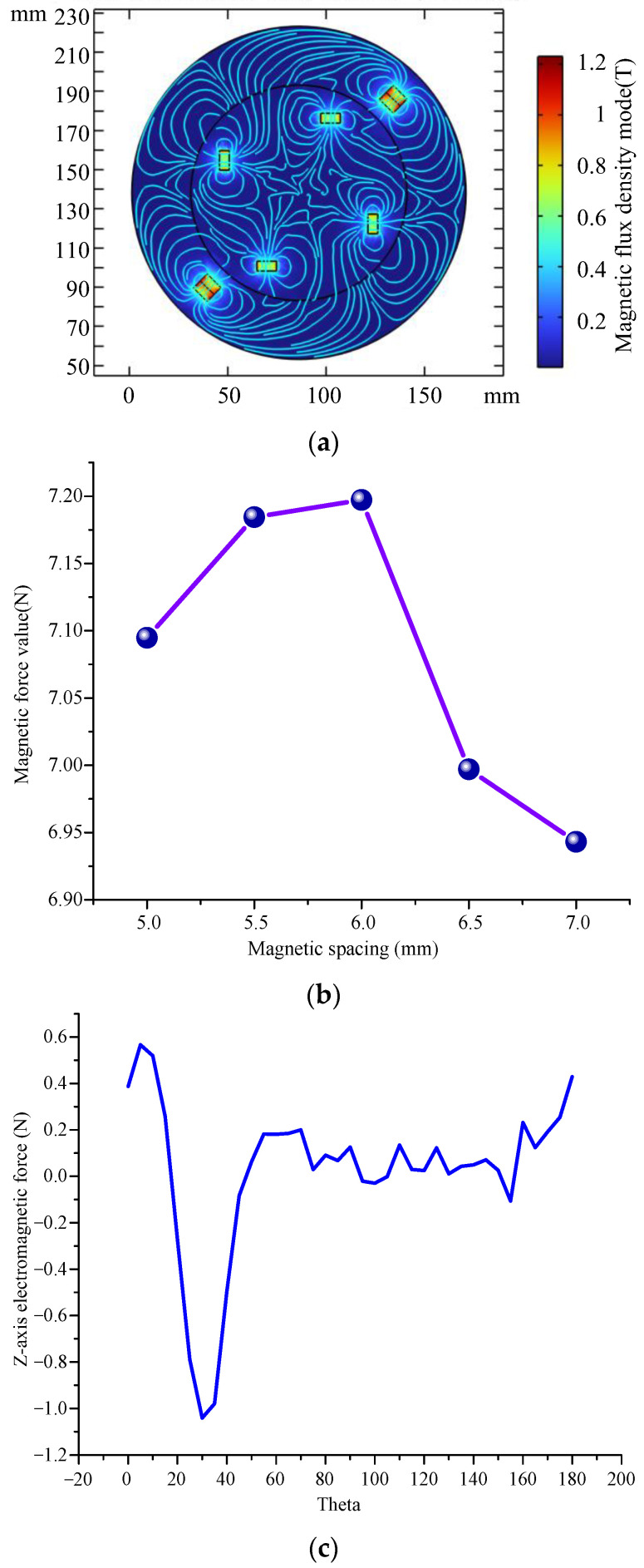

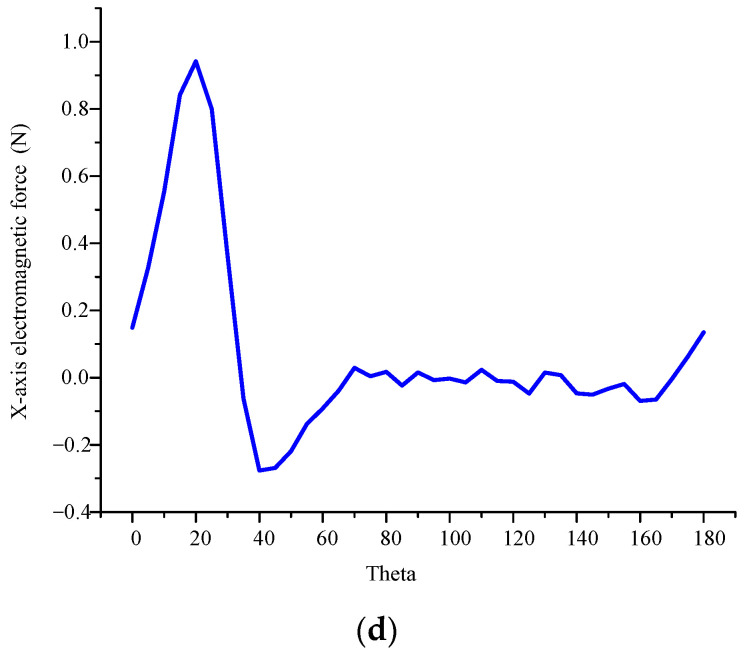
Simulation results of the magnetic field for the energy harvester. (**a**) Magnetic flux density distribution; (**b**) changes of magnetic force at different magnetic distances; (**c**) magnetic force of the piezoelectric cantilever along the *z* axis; (**d**) magnetic force of the piezoelectric cantilever along the *x* axis.

The simplified magnet model of energy harvester was imported into COMSOL software, and the magnetic fields generated by different magnets were simulated and analyzed. Figure 5 shows the simulation results of the magnetic field for the proposed energy harvester, where Figure 5a presents the density distribution of different magnets in the energy collector, Figure 5b gives the changes of magnetic force at different magnetic distances, while Figure 5c,d present the magnetic force of the piezoelectric cantilever along the *z* and *x* axis.

The results show that the maximum magnetic flux density is located at the two square magnets in the side slot of the rotor, which can deform the piezoelectric flexible sheet on the stator when the rotor rotates. In addition, the other four points with higher flux density are located at the bottom of the rotor at the circular magnet, which acts to make the coil in a changing magnetic field to generate electric charges. When the cantilever beam is subjected to the magnetic force of the permanent magnet, the cantilever beam will deform and the magnetic distance between the permanent magnet at the end of the cantilever beam and the permanent magnet on the rotor will change. When the magnetic distance is 6 mm, the force between permanent magnets is the largest, and the cantilever beam produces the largest deformation. Furthermore, the electromagnetic force on the cantilever beam has a maximum value in the z direction and the x direction in one period. The reason is that the maximum electromagnetic force is generated only when the magnet is closest to the cantilever beam in a period, which is consistent with the actual situation.

## 4. Experimental Analysis

**Figure 6:**
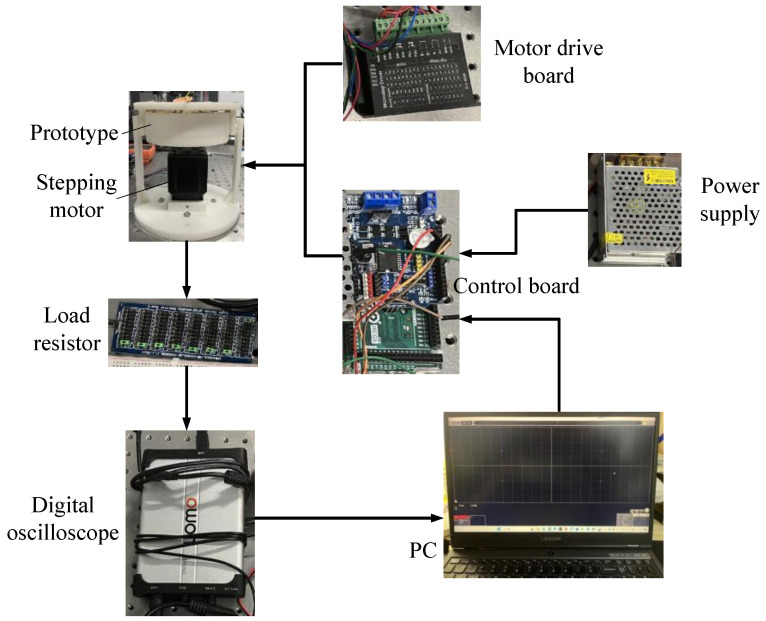
Experimental test system of the proposed energy harvester.

To verify the output performance of the piezoelectric energy harvester proposed in this paper, an experimental test system was built, as shown in Figure 6. In the test system, a stepping motor is used to apply rotational excitation to the energy harvester. The control board and motor drive board are used to apply driving signals to the stepping motor. In addition, digital oscilloscope and computer are used to obtain voltage data generated by the energy harvester.

**Figure 7:**
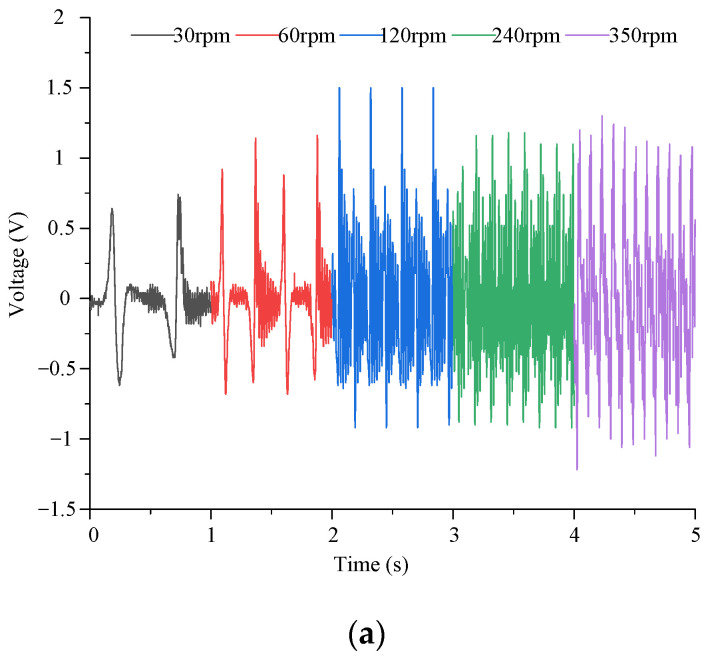

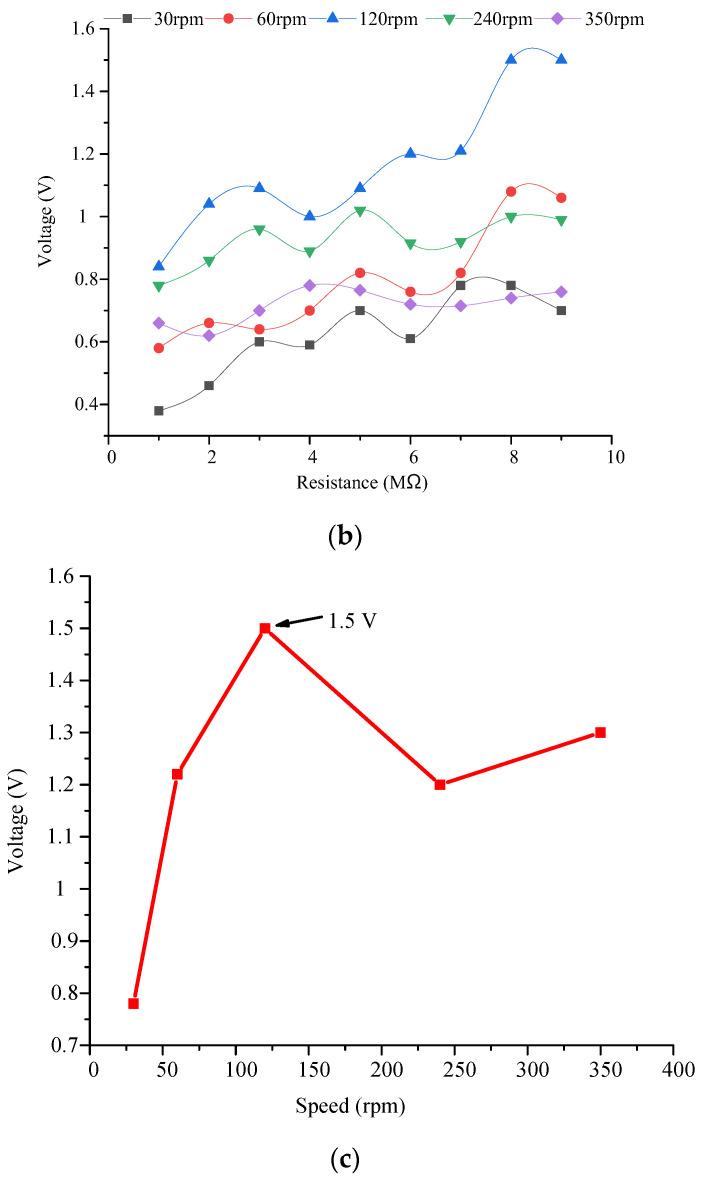
Output voltage test results of the piezoelectric module for the energy harvester. (**a**) The output voltage varies with time; (**b**) the output voltage varies with the load resistance; (**c**) the output voltage varies with the excitation speed.

Firstly, the output energy of the piezoelectric module of the energy harvester is tested experimentally. The piezoelectric MFC output wire is connected to the load resistance terminal, and the load resistance is connected to the virtual oscilloscope. The voltage signal generated by the energy collector is collected by the virtual oscilloscope. The output voltage experimental results are shown in Figure 7.

The output voltage increases first and then begins to decrease with the increase of excitation speed. The reason is that the output voltage of piezoelectric MFC has a certain limit. At the beginning, with the increase of the excitation speed, the output characteristics of the piezoelectric material gradually reach the best state. When the piezoelectric material reaches the optimal output voltage state, if the excitation speed continues to increase, the deformation of the piezoelectric material does not return to the initial state and then deforms in the next cycle, resulting in a decrease in output voltage.

When the excitation speed is constant, the output voltage changes steadily with time. As the load resistance increases, the output voltage tends to increase. However, the output voltage variation law has a large fluctuation. It is caused by many reasons. On the one hand, the output of the system is unstable due to the machining error and assembly error of the prototype. On the other hand, there is a certain error in the fit degree of each piezoelectric film to the cantilever beam. In addition, there are some differences in the magnet force received by each cantilever beam, resulting in errors in the output voltage under different working conditions.

When the load resistance is greater than 8 MΩ, the output voltage of the piezoelectric module shows a stable change. With the increase of excitation speed, the output voltage increases first and then decreases. When the driving speed is 120 rpm, the output voltage of the piezoelectric module reaches the maximum 1.5 V.

**Figure 8:**
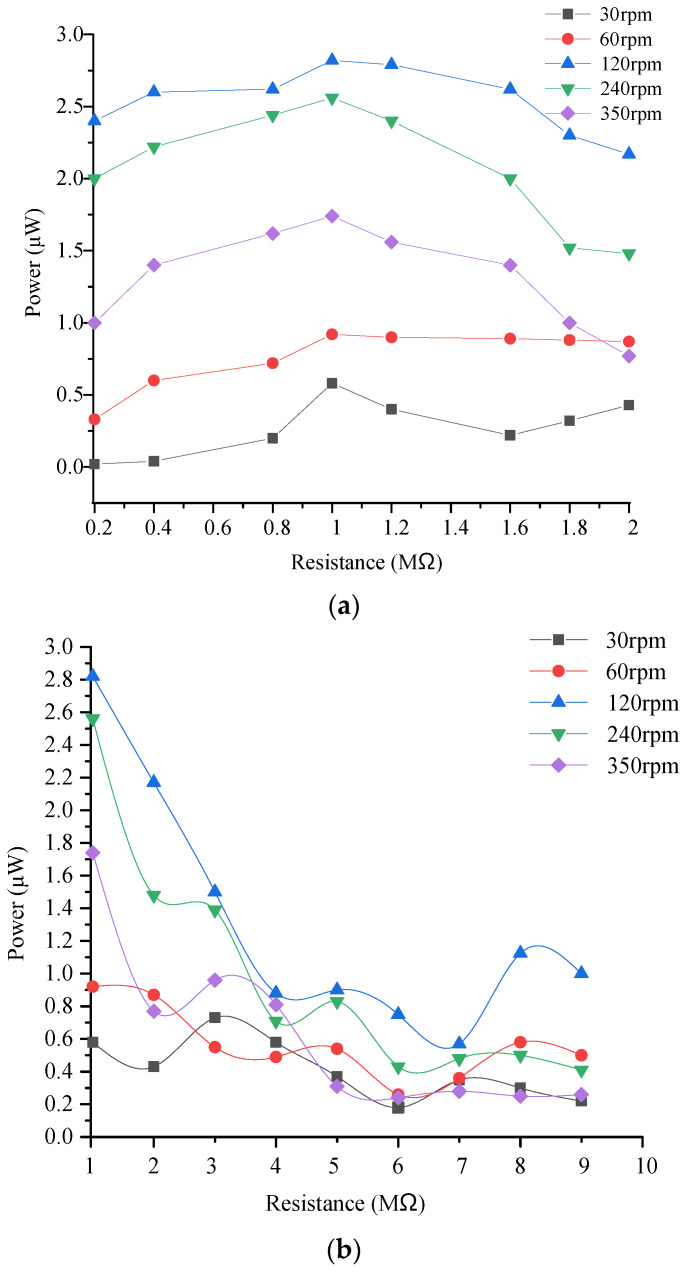
Output power test results of the piezoelectric module for the energy harvester. (**a**) Load resistance from 0 to 2 MΩ; (**b**) load resistance from 1 to 10 MΩ.

The output power of the piezoelectric module is tested, as shown in Figure 8. Figure 8a,b show the load resistance from 0 to 2 MΩ and from 1 to 10 MΩ, respectively. The results show that the variation trend of output power with load resistance is the same under different driving speeds. When the load resistance changes from 0–2 MΩ, the output power increases first and then decreases. When the load resistance reaches 1 MΩ, the output power at different speeds reaches the maximum value. When the load resistance is greater than 2 MΩ, the output power decreases first and then becomes stable. When the driving speed is 120 rpm and the load resistance is 1 MΩ, the output power of the piezoelectric module is 2.85 μW.

**Figure 9:**
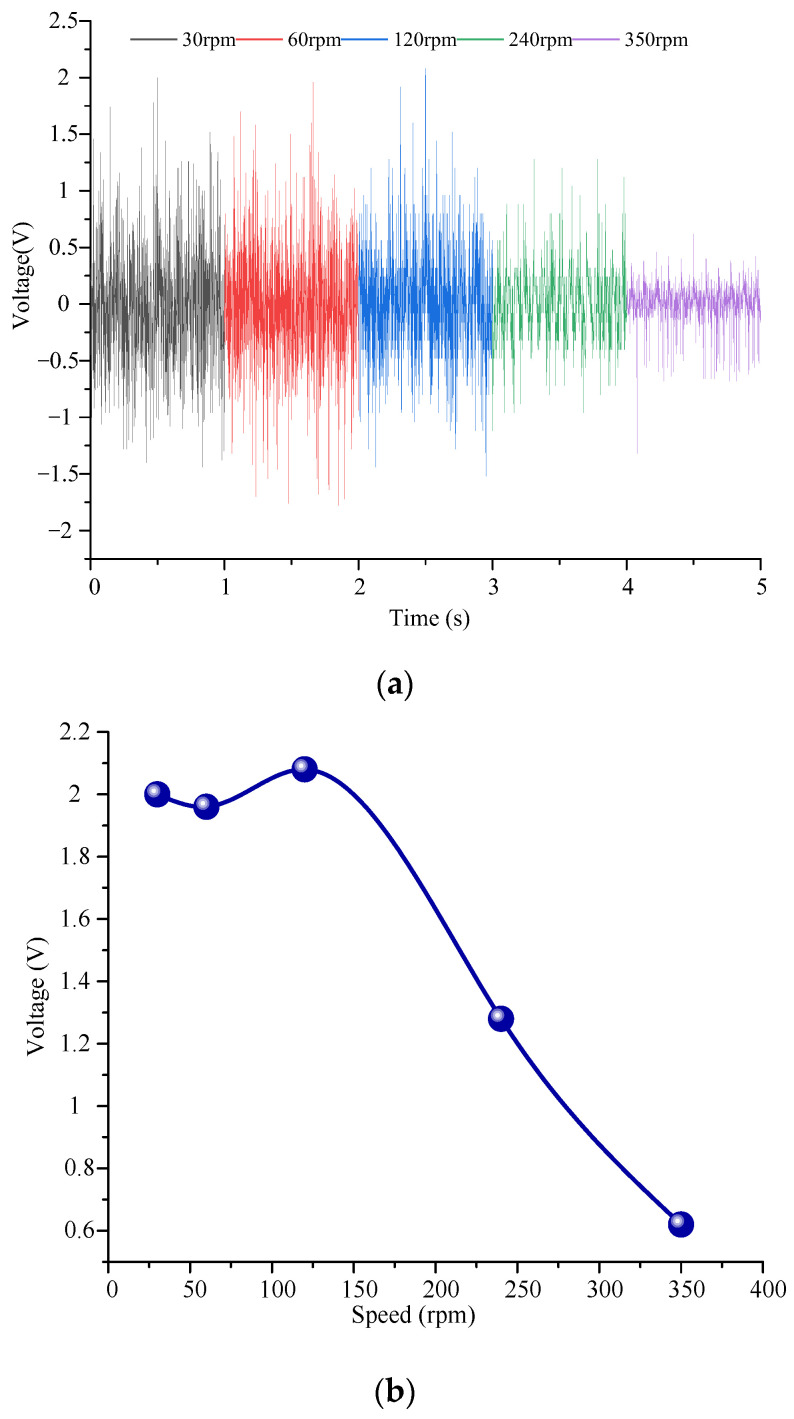
Output voltage test results of the electromagnetic module for the energy harvester. (**a**) The output voltage varies with time; (**b**) the output voltage varies with the excitation speed.

**Figure 10:**
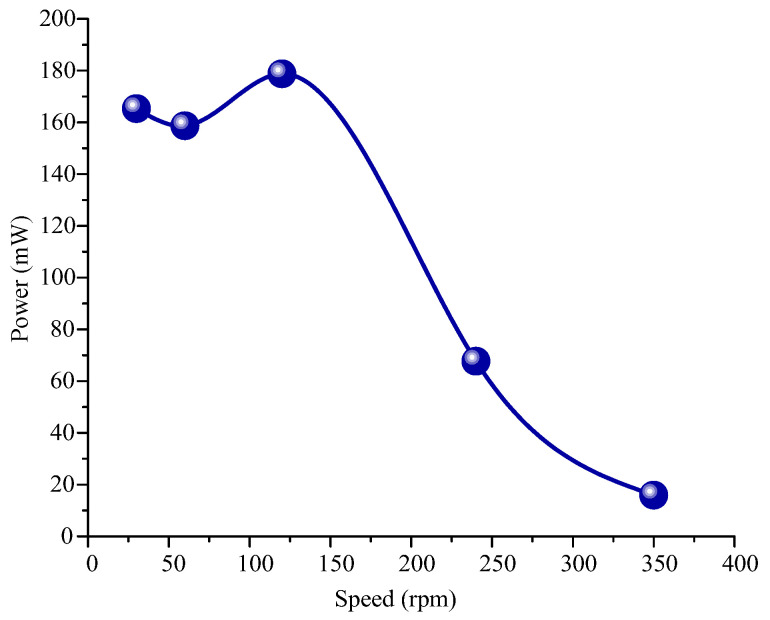
Output power test results of the electromagnetic module for the energy harvester.

In addition, to verify the output characteristics of the electromagnetic module, the output experiment of the electromagnetic module was carried out. During the test, the four coils of the electromagnetic module had been welded in series together, and the internal resistance measured via a multimeter is 24.2 Ω. The wire leading of the coil is connected with the conductive slip ring to ensure that the normal value can be measured during rotation and to avoid winding phenomenon. Then, the conductor of the conductive slip ring is connected to the computer, and the voltage under different speeds is obtained through the virtual oscilloscope. Finally, the power is obtained via Equation *P* = *U*^2^/*R*. Figure 9 and Figure 10 show the output voltage and power test results of the electromagnetic module for the energy harvester. The results show that:

(1) With the increase in the driving speed, the output voltage of the electromagnetic module increases first and then decreases. The maximum output voltage is 2.1 V when the driving speed is 120 rpm.
(2) The variation law of output power with speed is the same as that of voltage with speed. At the excitation speed of 120 rpm, the maximum output power can be achieved by 182 mW. After calculation, the maximum current at this time is 86.7 mA.
(3) Compared with the piezoelectric module, the output power of the electromagnetic module is much larger. When the piezoelectric module and electromagnetic module generate the maximum power, the excitation speed is different.

**Figure 11:**
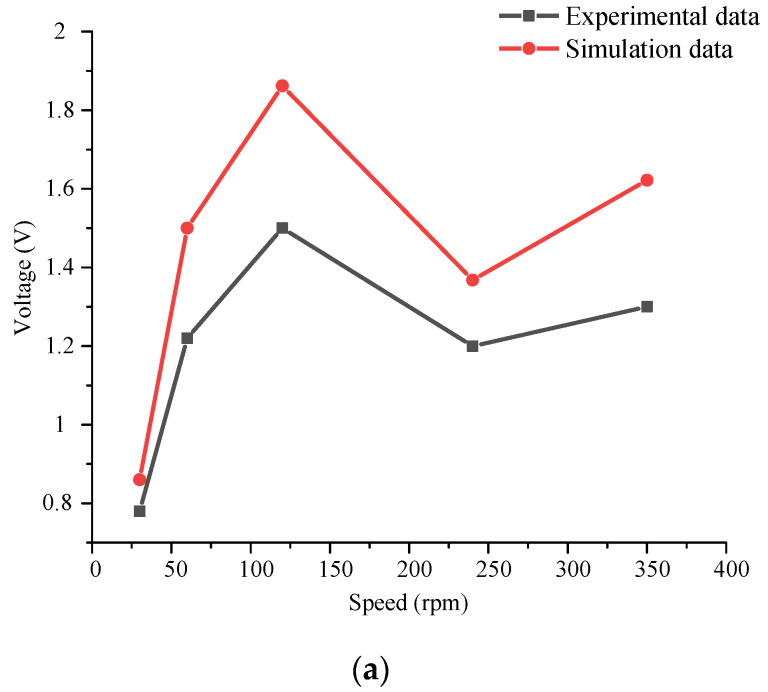

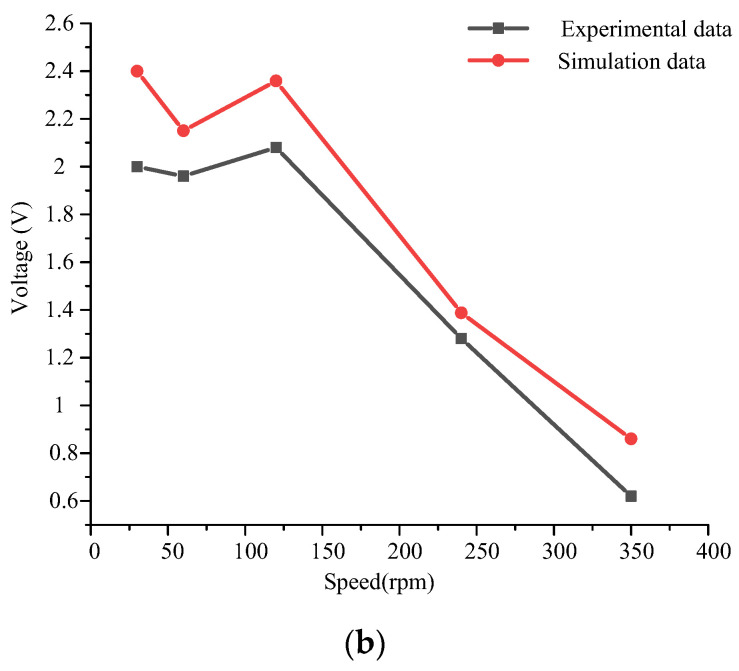
Comparison of experimental data with simulation data. (**a**) Comparison of output voltage of piezoelectric module; (**b**) comparison of output voltage of electromagnetic module.

**Figure 12:**
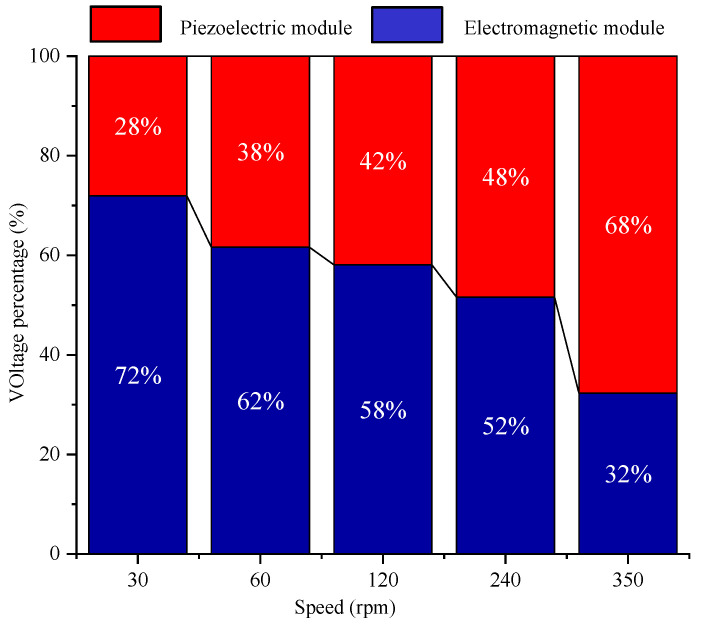
Comparison of the energy harvesting ratio of electromagnetic module and piezoelectric power module at different speeds.

To verify the correctness of the simulation model, the output simulation results of the piezoelectric module and the electromagnetic module were compared with the experimental results, as shown in Figure 11. At the same time, to analyze the energy harvesting effect of each module under different excitation speed, the energy harvesting ratio of the piezoelectric module and electromagnetic module was compared, as shown in Figure 12. In Figure 12, the output voltage of both the piezoelectric module and the electromagnetic module is the open circuit voltage without load.

The results show that the variation law of output voltage with excitation speed is the same under experiment and simulation. The error between experimental results and simulation results is small in the piezoelectric module and the electromagnetic module. According to the comparison of the energy harvesting ratio of the electromagnetic module and the piezoelectric power module at different speeds, when the excitation speed is small, the output voltage of the electromagnetic module is larger, and the output voltage value of the piezoelectric module is smaller. With the increase of the excitation speed, the output voltage value of the piezoelectric module increases, and gradually exceeds the voltage value of the electromagnetic module.

Therefore, to obtain the best output effect of the piezoelectric–electromagnetic hybrid energy harvester proposed in this paper, a reasonable excitation speed should be set.

## 5. Conclusions

In this paper, a novel piezoelectric and electromagnetic energy harvester used for rotary excitation was developed, based on array magnetic coupling. The working principle of this kind of energy harvester was analyzed. The performance of the prepared harvester was evaluated in a home-made experimental platform. The results have shown that:

(1) At the excitation speed of 120 rpm, the maximum output power of the electromagnetic module for the proposed energy harvester reaches 182 mW.
(2) Both the piezoelectric module and the electromagnetic module can reach the maximum output power at the excitation speed of 120 rpm.
(3) The output voltage characteristics of the electromagnetic module are better when the excitation speed is low, while the output voltage characteristics of the piezoelectric module are better when the excitation speed is high.

## Figures and Tables

**Table 1:** Parameters of the piezoelectric power generation module.

Item	Values	Item	Values
MFC Type	M2807-P2	Brand	XMT
MFC thickness	0.3 mm	MFC effective working length	28 mm
Cantilever material	304 stainless steel	MFC effective working width	7 mm
Cantilever length	40 mm	MFC total length	37 mm
Cantilever width	16 mm	MFC total width	10 mm
Cantilever thickness	0.2 mm	MFC capacitance	20 nF

**Table 2:** Parameters of the electromagnetic power module.

Item	Values	Item	Values
Coil inside diameter	6 mm	Turns per coil	460
Coil outside diameter	25 mm	Coil impedance	24.2 Ω
Copper wire diameter	0.3 mm	Magnet diameter	20 mm
Coil thickness	5 mm		

## Data Availability

All data that support the findings of this study are included within the article.
